# Acute Surgery vs Conservative Treatment for Traumatic Acute Subdural Hematoma

**DOI:** 10.1001/jamanetworkopen.2025.35200

**Published:** 2025-10-03

**Authors:** Thomas A. Van Essen, John K. Yue, Jason Barber, Hester F. Lingsma, Dana Pisică, Hugo F. den Boogert, Jeroen T. van Dijck, Wouter A. Moojen, Peter Hutchinson, Amy J. Markowitz, Ewout W. Steyerberg, David O. Okonkwo, Yelena G. Bodien, Alex B. Valadka, Ramon Diaz-Arrastia, Claudia S. Robertson, Brandon Foreman, Vincent Y. Wang, Michael A. McCrea, Joseph T. Giacino, Esther L. Yuh, Godard C. W. de Ruiter, Nancy R. Temkin, Andrew I. R. Maas, Wilco C. Peul, Geoffrey T. Manley

**Affiliations:** 1 University Neurosurgical Center Holland, Leiden University Medical Center, Haaglanden Medical Center & Haga, Leiden–The Hague, the Netherlands; 2 Department of Neurosurgery, Leiden University Medical Center, Leiden–The Hague, the Netherlands; 3 Department of Neurological Surgery, University of California, San Francisco; 4 Brain and Spinal Injury Center, Zuckerberg San Francisco General Hospital, San Francisco, California; 5 Department of Neurological Surgery, University of Washington, Seattle; 6 Center for Medical Decision Sciences, Department of Public Health, Erasmus MC–University Medical Center Rotterdam, Rotterdam, the Netherlands; 7 Division of Neurosurgery, Department of Clinical Neurosciences, University of Cambridge and Addenbrooke’s Hospital, Cambridge, United Kingdom; 8 NIHR Global Health Research Group on Neurotrauma, University of Cambridge, Cambridge, United Kingdom; 9 Department of Biomedical Data Sciences, Leiden University Medical Center, Leiden, the Netherlands; 10 Julius Center, University Medical Center Utrecht, Utrecht, the Netherlands; 11 Department of Neurological Surgery, University of Pittsburgh, Pittsburgh, Pennsylvania; 12 Department of Surgery, Vanderbilt University Medical Center, Nashville, Tennessee; 13 Department of Physical Medicine & Rehabilitation, Vanderbilt University Medical Center, Nashville, Tennessee; 14 Department of Neurological Surgery, Vanderbilt University Medical Center, Nashville, Tennessee; 15 Department of Neurological Surgery, University of Texas Southwestern Medical Center, Dallas; 16 Traumatic Brain Injury Clinical Research Center, Department of Neurology, University of Pennsylvania, Philadelphia; 17 Department of Neurological Surgery, Baylor College of Medicine, Houston, Texas; 18 Department of Neurology, University of Cincinnati, Cincinnati, Ohio; 19 Department of Rehabilitation Medicine, University of Cincinnati, Cincinnati, Ohio; 20 Department of Neurological Surgery, Dell Medical School, University of Texas–Austin; 21 Department of Neurological Surgery, Medical College of Wisconsin, Milwaukee; 22 Department of Physical Medicine and Rehabilitation, Spaulding Rehabilitation Hospital and Harvard Medical School, Boston, Massachusetts; 23 Department of Radiology and Biomedical Imaging, University of California, San Francisco; 24 Department of Biostatistics, University of Washington, Seattle; 25 Department of Neurological Surgery, Antwerp University Hospital, Edegem, Belgium; 26 Department of Translational Neuroscience, Faculty of Medicine and Health Science, University of Antwerp, Antwerp, Belgium.

## Abstract

**Importance:**

It is unclear whether performing surgery for most patients with an acute subdural hematoma (ASDH) and traumatic brain injury (TBI) is superior to conservative treatment.

**Objective:**

To compare the effectiveness of a strategy preferring acute surgical ASDH evacuation with one preferring initial conservative treatment.

**Design, Setting, and Participants:**

This comparative effectiveness study used data from February 1, 2014, to July 31, 2018, from the prospective observational Transforming Research and Clinical Knowledge in Traumatic Brain Injury Study, conducted at 18 Level 1 trauma centers in the US. The study included patients with nonpenetrating TBI presenting to the emergency department and admitted within 24 hours after injury with ASDH detected on acute head computed tomography scan. Statistical analysis was performed from December 1, 2022, to December 20, 2024.

**Exposures:**

Acute surgical hematoma evacuation vs initial conservative treatment, comparing outcomes between centers according to treatment preferences, measured by the case mix–adjusted probability of undergoing acute surgery (vs conservative treatment) per center.

**Main Outcomes and Measures:**

Functional disability at 6 months was assessed with the Glasgow Outcome Scale–Extended at 6 months, analyzed with ordinal logistic regression adjusted for prespecified confounders, quantified with a common odds ratio (OR). Variation in center preference was quantified with a median OR (MOR).

**Results:**

Of 2697 included patients, 711 (mean [SD] age, 46.5 [19.4] years; 539 men [76%]) had an ASDH, of whom 148 (21%) underwent acute cranial surgery and 563 (79%) underwent initial conservative treatment. The acute surgery cohort had lower mean (SD) Glasgow Coma Scale scores (6.8 [4.4] vs 11.4 [4.6]), more pupil abnormalities (both pupils unreacting: 43 of 133 [32%] vs 41 of 477 [9%]), and fewer isolated ASDHs (eg, more with concurrent intracranial lesions; 92 of 133 [69%] vs 297 of 563 [53%%]) compared with the conservative treatment cohort. In the surgical cohort, 129 of 148 patients (87%) underwent decompressive craniectomy (DC), and 17 of 148 (11%) underwent craniotomy. In the conservative treatment cohort, 67 of 563 patients (12%) underwent delayed cranial surgery (DC or craniotomy). The proportion of patients undergoing acute surgery ranged from 0% to 86% (median, 17% [IQR, 5%-27%]) between centers, with up to a 3-fold higher probability of prognostically similar patients receiving acute surgery in one center compared with another random center (MOR, 2.95 [95% CI, 1.79-7.47]; *P* = .06). Center preference for acute surgery over initial conservative treatment was not associated with a better outcome (OR, 1.05 [95% CI, 0.88-1.26] per 22% [IQR, 5%-27%] increase in acute surgery at a given trauma center).

**Conclusions and Relevance:**

In this comparative effectiveness study, similar patients with traumatic ASDH were treated differently due to center-specific treatment preferences. Outcomes were similar in centers preferring surgical evacuation and those preferring initial conservative treatment. This study suggests that, for a patient with ASDH for whom a neurosurgeon experiences clinical equipoise between acute surgery vs (initial) conservative treatment, conservative treatment may be considered.

## Key Points

**Question:**

Among US trauma centers, is a treatment strategy for traumatic acute subdural hematoma (ASDH) preferring acute surgery superior to one preferring conservative treatment?

**Findings:**

In this comparative effectiveness study of 711 patients with ASDH across 18 trauma centers, treatment varied highly between centers; proportions of patients undergoing acute surgery ranged from 0% to 86% (median, 17% [IQR, 5%-27%]). Center preference for acute surgery over conservative treatment was not associated with better 6-month outcomes.

**Meaning:**

This study suggests that patients with ASDH treated in centers that prefer acute surgery over conservative treatment have outcomes similar to patients treated in centers that prefer conservative treatment over acute surgery.

## Teaser

This comparative effectiveness study assesses whether acute surgery for traumatic acute subdural hematoma is superior to initial conservative treatment.

## Introduction

Acute subdural hematoma (ASDH) is a common pathologic finding in traumatic brain injury (TBI) and is associated with significant mortality and long-term morbidity.^1^ Treatment options include immediate cranial surgery for evacuation or initial conservative (nonsurgical) medical management with the potential for operative intervention in the event of neurologic decompensation. For this decision, the neurosurgeon weighs surgical risks against the risk of early death or disability due to deterioration. In cases of rapid neurologic decline due to ASDH with mass effect, acute surgery is required to relieve intracranial hypertension or herniation or to prevent death.^2,3^ However, in most cases, deciding between surgery and conservative management is less clear.

The 2006 Brain Trauma Foundation Guidelines^4^ recommend surgery when the hematoma is significantly large, irrespective of neurologic condition, or in the case of progressive neurologic deterioration referable to the lesion. However, the supporting evidence for surgery is limited, derived primarily from noncomparative studies with a small study population.^5,6,7,8,9,10^ Consequently, the threshold for ASDH surgical evacuation varies substantially among centers,^5,6,7^ reflecting strong center-specific treatment preferences that may signal clinicians’ uncertainty.^8,9^

Practice variations offer opportunities to assess the clinical effectiveness of interventions by examining the association between treatment variation and patient outcomes.^9^ In the recent large observational cohort studies Collaborative European NeuroTrauma Effectiveness Research in Traumatic Brain Injury (CENTER-TBI; conducted in Europe and Israel) and Transforming Research and Clinical Knowledge in Traumatic Brain Injury (TRACK-TBI; conducted in the US), designed for comparative effectiveness research, preferred local treatment approaches were embraced to estimate their clinical effectiveness.^11^ CENTER-TBI previously reported that substantial practice variations in acute surgery vs initial conservative treatment for ASDH were not associated with differences in outcome.^12^ We aimed to replicate this study in the US using TRACK-TBI. Our predefined goal was to compare the effectiveness of acute surgical evacuation with that of initial conservative treatment for patients with TBI and ASDH.

## Methods

The TRACK-TBI study is registered with ClinicalTrials.gov (NCT02119182). The protocol of the current analysis was approved by the TRACK-TBI Executive Committee prior to initiating data analysis. The analytical method conformed to the published CENTER-TBI (ClinicalTrials.gov NCT02210221) method^11,12^ and was conducted by the TRACK-TBI statistical team. The analysis followed the Strengthening the Reporting of Observational Studies in Epidemiology (STROBE) reporting guideline and statement using instrumental variable analysis recommendations,^13,14^ and corresponded to IDEAL (Idea, Development, Exploration, Assessment, Long-term study) Framework Stage 3.^15^ The institutional review board at each TRACK-TBI site approved all study protocols. Patients or their legally authorized representatives provided written informed consent. The Galveston Orientation and Amnesia Test assessed patient capacity to independently provide consent.^16^ For patients without a passing score, the patient’s legally authorized representative provided initial consent. Competency screening was repeated at each follow-up visit, and if a passing score was achieved, the patient was reconsented for study participation. Detailed study protocols, consent forms, and data collection forms are available online.^17^ Secondary data analyses using TRACK-TBI study data did not require additional institutional board review or informed consent procedures.

### Study Population

TRACK-TBI was a prospective, observational study across 18 US Level 1 trauma centers. Patients of all ages, without preexisting major neurologic or psychiatric disorders, who presented to the emergency department (ED) of a participating center within 24 hours of acute nonpenetrating TBI and completed a head computed tomography (CT) scan were eligible for enrollment. Traumatic brain injury was defined, per the American Congress of Rehabilitation Medicine Criteria, as a traumatically induced physiological disruption of brain function, such as loss of consciousness, amnesia, alteration of mental status after the accident, and/or neurologic deficits.^18^ Patients considered by the medical team and the principal investigator of the participating center to be clinically brain dead or deemed to have an unsurvivable injury were not included. The enrollment period was from February 1, 2014, to July 31, 2018, by convenience sampling. All Level 1 trauma centers that participate in TRACK-TBI provided patients who were included in the study population.

The current study is a secondary analysis of the TRACK-TBI dataset. We excluded patients younger than 17 years and those discharged directly from the ED. We selected patients with ASDH regardless of size or presumed necessity for surgical or nonsurgical conservative (medical management) treatment. To comprehensively compare treatment preferences, we intentionally included all patients with ASDH. However, in the sensitivity analysis, we restricted the analytic sample to patients without an extreme prognosis on either end of the spectrum: specifically, those with 1 or 2 unreactive pupils (poorer prognosis) or with a Glasgow Coma Scale (GCS) score of 15 (relatively good prognosis) at ED arrival.

### Center Characteristics and Data Management

TRACK-TBI data were collected in accordance with National Institute of Neurological Disorders and Stroke TBI Common Data Elements (CDEs).^19^ Deidentified CT scans were transmitted to a central imaging repository (laboratory of neuroimaging) and coded in accordance with the TBI neuroimaging CDEs^20^ by a central board–certified neuroradiologist (E.L.Y.) blinded to clinical data. Data were collected by trained personnel using web-based case report forms (QuesGen Systems).^20^ The cranial surgery cohort has previously been described, including definitions of surgery and surgery-related variables.^21^

Several preparatory methodological studies were performed.^12,22,23^ Associations between acute care decisions for TBI, such as prehospital and intensive care, were quantified with the surgical threshold for ASDH. Using cluster analysis, it was reported that acute treatment preferences for TBI within centers were unrelated and the absence of correlation between domains was most pronounced for neurosurgery.^22^ Moreover, it was found that a priori acute surgery preferences were more strongly associated with surgery rates than the strongest TBI risk factors (age, GCS score, pupils, and CT scan abnormalities).^12,23^ This finding indicates that the instrumental variable used for the current analysis (ie, the case-mix–adjusted [observed] acute surgery rates) reflects the center treatment preference and not unmeasured case-mix differences.

### Interventions

Acute cranial surgery was defined as taking place less than 6 hours after the first head CT scan. Conservative treatment was defined as nonsurgical, medical management after the first CT scan, with potential delayed surgery (>6 hours after the first CT scan). Surgery indication, type, and timing of transfer to the operating room were evaluated. Surgical treatment was at the discretion of the treating neurosurgeon and consisted of ASDH evacuation by craniotomy or by a (primary) decompressive craniectomy (DC), defined as craniotomy without bone flap replacement to accommodate brain swelling. The initial conservative approach was defined as medical management after the first CT scan, with admission to the hospital ward or intensive care unit, inclusive of intracranial pressure monitoring and delayed surgical evacuation.

### Outcomes

The primary outcome was the score on the Glasgow Outcome Scale–Extended (GOSE), an 8-point scale ranging from 1 (death) to 8 (upper good recovery), at 6 months.^24^ The GOSE was administered by trained personnel to capture TBI-related disability (ie, excluding disability attributable to extracranial injuries). Secondary outcomes included in-hospital mortality, hospital length of stay (in days), discharge destination, and 6-month quality of life assessed with the Quality of Life after Brain Injury Questionnaire Overall Scale (Qolibri-OS).^25^ Measures were administered through structured interviews by trained personnel at each outcome time point.

### Statistical Analysis

Statistical analysis was performed from December 1, 2022, to December 20, 2024. Practice variation was described using proportions and IQRs of patients undergoing acute surgery per center. Between-center differences in acute surgery were adjusted for case mix and quantified with a median odds ratio (MOR),^26^ which depicts the treatment variation between centers not attributable to chance and not explained by other case-mix factors.

The main analysis associated center-level treatment strategies with functional outcome to derive effectiveness. This natural experiment (ie, patients are “allocated” to one or another treatment strategy based on where the accident occurred) may lead to reduction of unmeasured confounding because patients are brought to hospitals without knowledge of neurosurgical treatment preference (eFigure 1 in Supplement 1).^23,27,28^ Specifically, we compared centers with different preferences for acute surgical evacuation, quantified by the case-mix–adjusted probability of acute surgery vs initial conservative treatment as observed in each center. We presented baseline characteristics and CRASH-CT (Corticosteroid Randomisation After Significant Head Injury Trial–CT) scores, a validated baseline prognostic model integrating country, age, GCS score, pupil abnormality, major extracranial injury, and presence of CT scan abnormalities (petechial hemorrhages or diffuse axonal injury, basal cistern obliteration, subarachnoid hemorrhage, midline shift, and evacuated hematoma),^29^ across quartiles of the instrumental variable (ie, the case-mix–adjusted probability of performing acute surgery). The first quartile contains centers least likely to perform acute surgery, and the fourth quartile contains centers most likely to perform acute surgery. The instrumental variable analysis is based on preference for acute surgery rates as a continuous variable; the quartiles are presented to provide insight as to the comparability of patient populations across the instrument, which allows the reader to evaluate how comparable the patient characteristics are (instrumental variable assumption; the instrument is independent of confounders).^14,30^

The primary effect estimate was the adjusted common odds ratio (OR) for better outcome on the GOSE. This ratio was estimated with random-effects ordinal logistic regression with the instrument (adjusted probability of performing acute surgery) as a continuous variable. The random-effects term accounts for other between-center differences than the factors included in the model, thereby adjusting for center clustering of other care processes or hospital structures. Confounding was further addressed by adjusting for the predefined variables of age, GCS score, pupil reactivity, concomitant contusion, and midline shift.^11^ The common OR is presented as a comparison between the first and the fourth quartiles of the instrumental variable (the adjusted probabilities for undergoing acute surgery) and reflects the odds for a more favorable outcome when comparing centers preferring acute surgery over initial conservative treatment.

Analyses were performed in SAS, version 9.4 (SAS Institute Inc). Analyses used multiple imputation by chained equations to account for covariables presumed to be missing at random and inverse probability weighting by boosted regression to account for potential bias due to excluding patients with missing outcomes. Odds ratios and β coefficients are presented with 95% CIs and *P* values. We report the uncertainty in these estimates through 95% CIs, not by claiming significance or nonsignificance in the *P* values.

The main analyses, which were performed by an independent statistical team, were also conducted independently by a second team. The eMethods in Supplement 1 provides additional details of the methods and sensitivity analyses.^31^

## Results

### Patient Characteristics

Of 2697 patients with TBI in the TRACK-TBI study, 711 (mean [SD] age, 46.5 [19.4] years; 539 men [76%] and 172 women [24%]) had an ASDH (eTable 1 in Supplement 1). Of these 711 patients, 148 (21%) received acute surgery, and 563 (79%) received initial conservative treatment. The acute surgery cohort had lower ED arrival GCS scores (6.8 [4.4] vs 11.4 [4.6]), more pupil abnormalities (both pupils unreacting; 43 of 133 [32%] vs 41 of 477 [9%]), and fewer isolated ASDHs (eg, more with concurrent intracranial lesions; 92 of 133 [69%] vs 297 of 563 [53%%]) compared with the conservative cohort.

**Figure 1.**
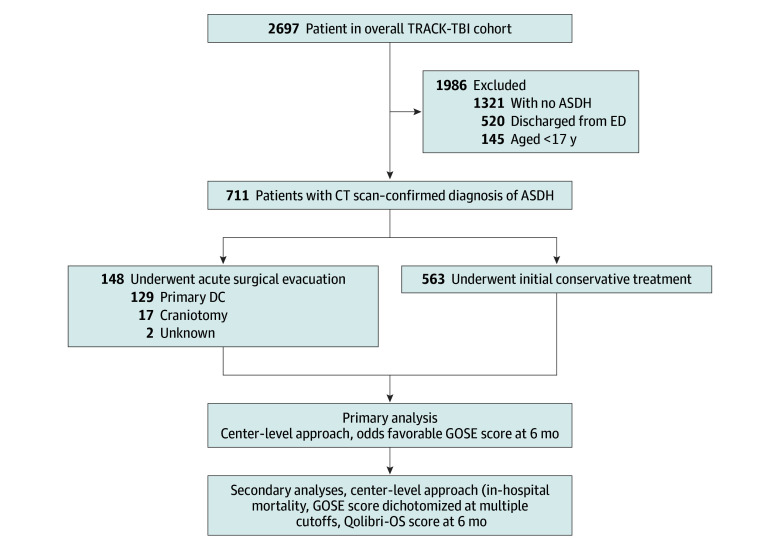
Flow Diagram of Study Population and Data Analyses ASDH indicates acute subdural hematoma; CT, computed tomography; DC decompressive craniectomy; ED, emergency department; GOSE, Glasgow Outcome Scale Extended; Qolibri-OS, Quality of Life after Brain Injury Overall Scale; and TRACK-TBI, Transforming Research and Clinical Knowledge in Traumatic Brain Injury.

Acute surgery was performed a median of 3.2 hours (IQR, 1.9-4.3 hours) after injury (eTable 1 in Supplement 1). In the conservative treatment cohort, 67 of 563 patients (12%) underwent delayed cranial surgery (DC or craniotomy). Of the 563 patients initially conservatively treated, 42 (7%) underwent delayed surgery for ASDH (DC or craniotomy) a median of 36.3 hours (IQR, 14.3-95.6 hours) after injury, while 21 patients (4%) underwent DC or craniotomy for indications other than ASDH. Of the 148 patients in the acute surgery cohort, 129 (87%) underwent primary DC, and 17 (11%) underwent craniotomy (Figure 1); 105 (71%) received intracranial pressure monitoring. Of the 563 patients in the conservative treatment cohort, 142 (25%) underwent intracranial pressure monitoring (eTable 2 in Supplement 1). Of patients with 2 nonreactive pupils, 51% (43 of 84) underwent acute surgery. Of 233 patients with an ED GCS score of 15, 222 (95%) received initial conservative treatment.

### Practice Variation

**Figure 2.**
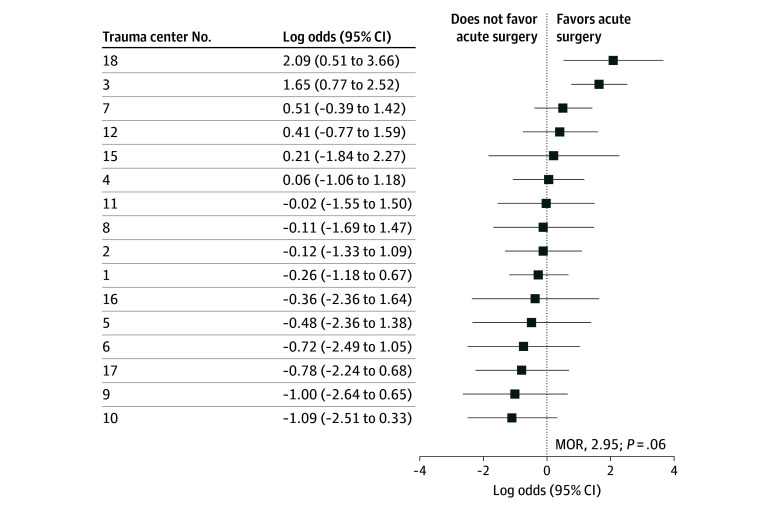
Between-Center Differences in Acute Surgery for Traumatic Acute Subdural Hematoma The x-axis presents the log odds of the adjusted acute surgery rates per center. A logistic random-effects model, adjusted for the predefined confounders of age, Glasgow Coma Scale score, pupil reactivity, concomitant contusion, and midline shift, was used to estimate acute surgery preference per center with corresponding 95% CIs. MOR indicates median odds ratio.

The observed proportion of patients undergoing acute surgery ranged from 0% to 86% (median, 17% [IQR, 5%-27%]) between centers (eTable 3 in Supplement 1). The associated MOR for acute surgery was 2.95 (95% CI, 1.79-7.47) (*P* = .06), reflecting a 3-fold higher probability of undergoing acute surgery for a similar patient in one center vs another random center (Figure 2).

The proportion of acute surgery ranged from 0% to 100% (median, 20% [IQR, 5%-34%]) for patients with 2 reactive pupils and an ED GCS score less than 15. For patients with an ED GCS score of 15, the proportion of acute surgery ranged from 0% to 20% (median, 2% [IQR 0%-6%]). For patients with 2 nonreactive pupils, the proportion of acute surgery ranged from 0% to 100% (median, 53% [IQR, 46%-79%]); for patients with 1 nonreactive pupil the proportion of acute surgery ranged from 0% to 67% (median, 16% [IQR, 7%-30%).

The most important confounders and baseline prognosis (ie, estimated 6-month functional outcome at baseline on the CRASH-CT score) were similar across centers ranked by acute surgery preferences for ASDH (Table 1), which reflected a balance in patient populations across the instrumental variable analysis. Findings were consistent when analyses were restricted to patients with 2 reactive pupils and a GCS score less than 15 (eTable 4 in Supplement 1). Formally, the testable assumptions for instrumental variable analyses were met (eResults in Supplement 1). Hence, the considerable variations in treatment approaches stem from centers that, on average, cater to prognostically similar patients.

**Table 1.** Baseline Characteristics and Prognostic Risk Across Trauma Centers With Different Preferences for Immediate Surgery of Acute Subdural Hematoma

Characteristic	Acute surgery preference (case-mix–adjusted random-effects coefficient from mixed model)^a^				*r* Value^b^	*P* value^c^
	Quartile 1 (<−0.50) (n = 103)	Quartile 2 (−0.50 to −0.25) (n = 199)	Quartile 3 (−0.25 to 0.25) (n = 140)	Quartile 4 (>0.25) (n = 269)		
Age, median (IQR), y	48 (31-64)	45 (27-57)	46 (28-60)	47 (32-64)	0.04	.25
Sex, No. (%)						
Female	19 (18)	50 (25)	39 (28)	64 (24)	NA	.01
Male	84 (82)	149 (75)	101 (72)	205 (76)		
Race, No. (%)						
Asian	5 (5)	6 (3)	4 (3)	26 (10)	NA	<.001
Black	12 (13)	32 (17)	21 (15)	13 (5)		
White	74 (80)	152 (79)	112 (80)	224 (84)		
Years of education, median (IQR)	12 (12-16)	12 (11-14)	12 (12-14)	13 (12-16)	0.17	<.001
Working (before injury), No. (%)	53 (63)	115 (69)	88 (64)	151 (61)	NA	.42
Married or domestic partnership, No. (%)	45 (57)	79 (46)	56 (41)	108 (44)	NA	.13
Uninsured, No. (%)	22 (27)	55 (30)	22 (16)	26 (10)	NA	<.001
History of cardiovascular disease, No. (%)	22 (21)	47 (24)	34 (24)	85 (32)	NA	.11
History of neurologic disease, No. (%)	12 (12)	13 (7)	14 (10)	44 (16)	NA	.01
History of oncologic disease, No. (%)	6 (6)	5 (3)	7 (5)	11 (4)	NA	.44
History of endocrine disease, No. (%)	17 (17)	36 (18)	33 (24)	61 (23)	NA	.35
Population type, No. (%)						
Rural	2 (2)	6 (3)	4 (4)	5 (2)	NA	.55
Micropolitan	3 (3)	2 (1)	3 (3)	14 (6)		
Metropolitan	91 (95)	176 (9)	103 (94)	225 (92)		
Injured by acceleration or deceleration, No. (%)	26 (25)	51 (26)	60 (43)	127 (47)	NA	.20
Alcohol use (>2 drinks/d), No. (%)	31 (41)	54 (33)	47 (34)	89 (40)	NA	.02
Cause of injury, No. (%)						
Road traffic incident	39 (39)	86 (44)	62 (45)	140 (52)	NA	.07
Incidental fall	44 (44)	66 (34)	53 (38)	93 (35)		
Other	17 (17)	44 (22)	24 (17)	35 (13)		
Transferred from other hospital, No. (%)	27 (26)	49 (25)	41 (29)	61 (23)	NA	.51
Arrival method, No. (%)						
Helicopter	23 (22)	31 (16)	40 (29)	89 (33)	NA	<.001
Ground ambulance with physician	14 (14)	1 (1)	4 (3)	1 (0)		
Ground ambulance no physician	62 (60)	160 (80)	95 (68)	173 (65)		
Intubation in field or in ED, No. (%)	29 (28)	49 (25)	28 (20)	87 (33)	NA	.05
Hypoxia, No. (%)	15 (15)	10 (5)	18 (13)	18 (7)	NA	.006
Hypotension, No. (%)	13 (13)	17 (9)	7 (5)	30 (11)	NA	.12
Any major extracranial injury, No. (%)^d^	17 (19)	46 (23)	25 (18)	48 (18)	NA	.58
Baseline GCS score, mean (SD)	10 (5)	11 (5)	11 (5)	10 (5)	−0.03	.39
Baseline GCS motor score, mean (SD)	4 (2)	5 (2)	5 (2)	4 (2)	0.01	.89
Pupils, No. (%)						
Both reacting	68 (76)	137 (81)	92 (77)	197 (85)	NA	.16
One reacting	4 (4)	10 (6)	8 (7)	10 (4)		
Both unreacting	17 (19)	22 (13)	20 (17)	25 (11)		
Anticoagulants or platelet aggregation inhibitors, No. (%)						
None	60 (80)	164 (88)	117 (86)	192 (79)	NA	.02
Anticoagulants	0	0	4 (3)	4 (2)		
Platelet inhibitors	13 (17)	22 (12)	15 (11)	42 (17)		
Both	2 (3)	0	0	5 (2)		
Time from injury to CT scan, median (IQR), h	1.7 (1.1-3.3)	1.3 (0.9-2.4)	1.5 (1.0-2.4)	1.6 (1.0-2.7)	−0.06	.10
CT scan of head, No. (%)						
Midline shift ≥5 mm	17 (17)	34 (18)	23 (17)	46 (17)	NA	>.99
Contusion	48 (49)	115 (59)	71 (51)	155 (58)		.21
Subarachnoid hemorrhage	77 (79)	151 (78)	96 (70)	213 (80)		.12
Shear	12 (12)	30 (15)	13 (9)	54 (20)		.03
Basal cisterns absent or compressed						
No effacement	70 (71)	143 (74)	100 (72)	185 (70)	NA	.77
Partial effacement	14 (14)	33 (17)	27 (20)	53 (20)		
Complete effacement	14 (14)	18 (9)	11 (8)	28 (11)		
CRASH estimated 6-mo unfavorable outcome (GOS score ≤3), median (IQR), %^e^	39 (12-72)	35 (14-52)	36 (16-61)	39 (15-66)	0.02	.69
Center characteristics						
Academic hospital (vs nonacademic), No. (%)	103 (100)	199 (100)	140 (100)	269 (100)	NA	NA
No. of beds, mean (SD)	812 (429)	574 (270)	592 (194)	661 (111)	−0.09	.02
Neurosurgery residency program, No. (%)	103 (100)	199 (100)	140 (100)	269 (100)	NA	NA
Level 1 trauma center designation, No. (%)	103 (100)	199 (100)	140 (100)	269 (100)	NA	NA
Urban location (vs suburban and rural location), No. (%)	103 (100)	199 (100)	140 (100)	269 (100)	NA	NA
Neurosurgeon staffing, mean (SD), FTE	8 (6)	8 (9)	7 (3)	9 (4)	0.07	.07

Abbreviations: CRASH, Corticosteroid Randomisation After Significant Head Injury; CT, computed tomography; ED, emergency department; FTE, full-time equivalent; GCS, Glasgow Coma Scale; GOS, Glasgow Outcome Scale; NA, not applicable.

^a^ Treatment preference as defined by the case-mix–adjusted probability of undergoing acute surgery (as opposed to initial conservative treatment) based on the observed acute surgery rates per center. The first category is less aggressive than the second and the second is less aggressive than the third, and so forth. The instrumental variable analysis used the acute surgery rates as continuous preference; the quartiles are presented for purposes of interpretability of baseline comparability. Based on random-effect coefficient from mixed model.

^b^ Rank correlation coefficient by Spearman correlation based on the actual site-coefficient value.

^c^ *P* value; statistical significance by the Kruskal-Wallis and Fisher exact tests (no monotonicity assumption) of the categorical variables, or statistical significance of the Spearman correlation (monotonicity assumption).

^d^ Any nonhead or neck Abbreviated Injury Scale score of 3 or more.

^e^ Traumatic brain injury severity as summarized in estimated unfavorable outcome, proportion with a GOS score of 3 or less, based on CRASH-CT variables.

### Associations With Outcome

Center preference for acute surgery over initial conservative treatment was not associated with better outcomes (adjusted common OR, 1.05 [95% CI, 0.88-1.26] for more favorable 6-month outcome per 22% [IQR, 5%-27%] increase in acute surgery at a given trauma center) (Table 2; eResults and eFigure 2 in Supplement 1). The ORs were consistent across various GOSE dichotomizations (Table 2).

**Table 2.** Primary and Secondary Outcomes and Association With Acute Surgery

Outcome	Acute surgery preference (case-mix–adjusted random-effects coefficient from mixed model)				Effect variable	Adjusted value (95% CI)^a^
	Quartile 1 (<−0.50) (n = 103)	Quartile 2 (−0.50 to −0.25) (n = 199)	Quartile 3 (−0.25 to 0.25) (n = 140)	Quartile 4 (>0.25) (n = 269)		
Primary outcome: GOSE score at 6 mo, mean (SD)^b^	4 (3)	5 (2)	6 (2)	5 (3)	Common odds ratio	1.05 (0.88 to 1.26)
Secondary outcomes						
In-hospital mortality, No. (%)	21 (20)	12 (6)	13 (9)	33 (12)	Odds ratio	0.88 (0.65 to 1.20)
GOSE score of 7 or 8, No./total No. (%)	19/57 (33)	44/123 (36)	47/107 (44)	94/230 (41)	Odds ratio	1.09 (0.92 to 1.29)
GOSE score of 5-8, No./total No. (%)	32/57 (56)	81/123 (66)	77/107 (72)	146/230 (63)	Odds ratio	0.90 (0.74 to 1.11)
GOSE score of 4-8, No./total No. (%)	32/57 (56)	82/123 (67)	81/107 (76)	153/230 (67)	Odds ratio	0.94 (0.76 to 1.16)
Qolibri-OS score at 6 mo, median (IQR)^c^	63 (46-88)	63 (42-82)	63 (46-81)	67 (50-83)	β	1.16 (−1.05 to 3.37)

Abbreviations: GOSE, Glasgow Outcome Scale Extended; Qolibri-OS, Quality of Life after Brain Injury Scale Overall Scale.

^a^ Estimates from random-effects multivariable logistic regression with the instrument, adjusted center probability of performing acute surgery as random effects treatment variable. Confounding was furthermore addressed by adjusting for the predefined variables of age, Glasgow Coma Scale score, pupil reactivity, contusion (presence or absence), and midline shift (per 1-mm shift). The (common) odds ratios are presented as comparisons between the first quartile and the fourth quartile (IQR) of the instrument (the adjusted probabilities for undergoing acute surgery), reflecting the odds of a higher (ie, more favorable) GOSE score.

^b^ The GOSE is an 8-point scale ranging from 1 (death) to 8 (upper good recovery) that assesses global disability and recovery after traumatic brain injury by quantifying the functional independence. GOSE score was available for 517 patients (120 in acute surgery group and 397 in conservative treatment group) for the primary analysis. Inverse probability weighting by boosted regression was used to counteract the bias caused by excluding the 194 patients who did not have a GOSE score available.

^c^ The Qolibri-OS is a standardized health-specific quality of life measure specifically designed for and validated for outcome assessment among patients with brain injury. The instrument consists of 6 items assessing overall satisfaction with life (physical condition, cognition, emotions, function in daily life, personal and social life, and current situation and future prospects). It is a numerical scale with scores ranging from 0 to 100, with higher scores indicating a better quality of life. The Qolibri-OS score was available for 352 patients (42 in acute surgery group and 310 in conservative treatment group).

When repeating the analysis restricted to patients with bilateral reactive pupils and a GCS score less than 15, the OR remained consistent (adjusted common OR, 0.99 [95% CI, 0.81-1.22]) (eTable 4 in Supplement 1). Also, estimates were similar when additionally adjusting for cardiovascular history (OR, 1.05 [95% CI, 0.88-1.26]), for anticoagulant use (OR, 1.08 [95% CI, 0.94-1.24]), for timing injury to CT scan (OR, 1.05 [95% CI, 0.88-1.26]), for transfer status (OR, 1.06 [95% CI, 0.91-1.25]), for race and ethnicity (OR, 1.04 [95% CI, 0.88-1.24]), and with different temporal definitions of acute surgery (acute surgery defined as surgical evacuation within 24 hours of CT scan [n = 166]; MOR, 2.31 [95 CI, 1.52-5.41; *P* = .16] and OR, 1.08 [95% CI, 0.82-1.43]; acute surgery defined as any surgical evacuation during acute hospitalization [n = 191], MOR, 2.00 [95 CI, 1.40-4.12; *P* = .17] and OR, 1.06 [95% CI, 0.80-1.39]) (eResults, eFigure 3, and eTable 4 in Supplement 1). Finally, in sensitivity analyses, adjustment in multivariable regression and propensity score weighting yielded estimates suggestive of a negative association between surgery and outcome (eTable 4 in Supplement 1). Secondary outcomes did not differ between groups (Table 2; eTable 5 in Supplement 1).

## Discussion

We found that patients with traumatic ASDH with similar prognoses received different clinical management strategies across 18 US Level 1 trauma centers. Patients treated in centers that preferred acute surgery over (initial) conservative treatment had similar 6-month outcomes as patients treated in centers that preferred (initial) conservative treatment. Results were consistent for patients with bilateral reactive pupils and a GCS score of 3 to 14, a patient population with greater clinical practice variation. As expected,^23^ patient-level analysis with multivariable regression and propensity score weighting revealed poorer functional outcomes for acute surgery due to residual confounding.

A randomized clinical trial to answer this question would be ideal but is highly challenging.^8^ The RESCUE-ASDH (Randomized Evaluation of Surgery with Craniectomy for Patients Undergoing Evacuation of Acute Subdural Hematoma) trial, conducted among patients with ASDH, examined the surgical technique rather than indication.^32^ Observational studies using instrumental variable analysis offer a promising alternative to conducting randomized clinical trials.^23,27,33,34^ An instrumental variable analysis can, like randomization, reduce bias due to unmeasured confounding, which regression and propensity score methods are unable to do. Our study validity hinges on the suitability of the center treatment rate as an instrumental variable. Residual (regional) prognostic differences may still be associated with the observed practice variations. Our instrument was strongly correlated with acute surgery and not with baseline prognosis, supporting its reliability. Variability in ASDH surgery rates is likely associated with clinician preferences.^5,7,12,22^ To further elucidate the associations between ASDH treatment strategies within centers and TBI outcomes, adjustments were made for other between-center differences using random-effects modeling.^23^

The findings should be considered within the context of the methods used. Our instrumental variable analysis offers insights into whether patients’ outcomes would improve if centers altered their policies regarding a specific intervention, rather than estimating effects on individual patients.^35,36^ The instrumental variable design measures a center-level policy effect, not an individual-level treatment effect. Here, the instrument is the center treatment rate, serving as a proxy for the surgeon’s treatment preference. Given that the same patient may undergo surgery in one center but not in another, it naturally follows that multiple treatment options exist. These results pertain to patients for whom neurosurgeons may be in equipoise, meaning that more than 1 treatment option can be considered. Because equipoise varies across centers, it is challenging to identify which patients and to what extent uncertainty applies.^37^ Nonetheless, from our findings, when there is equipoise regarding the decision to evacuate or not, no difference in outcome due to a center’s treatment strategy is anticipated.

Surgical evacuation of ASDH is a fundamental indication for treatment of life-threatening neurologic deterioration.^2,3,38^ The current findings do not subtract from the well-established effect of acute evacuation of large ASDH in neurologically compromised patients. Beyond patients with brain herniation and unreactive pupils, surgical timing and indications may vary, as a substantial proportion of patients who undergo medical stabilization as part of initial conservative management have satisfactory outcomes.^39,40,41,42,43^ Our findings replicate and extend reports from CENTER-TBI and therefore provide robust evidence that for patients with an ASDH in which there is uncertainty among the neurosurgical community regarding the immediate surgical indication, conservative, nonsurgical treatment may be thoughtfully considered in accordance with institutional best practices, clinician-specific knowledge, and ongoing clinical progression,^44^ factoring in major clinical modifiers such as frailty, multiple trauma, and prior cerebral insults.^45,46,47^

### Strengths and Limitations

This study has some strengths. Complex analyses of complex datasets with multiple defensible analytic approaches are often subject to biases.^48^ Our study benefits from a comparative effectiveness design; predefined analyses; using robust, contemporary, prospective and standardized data corroborated by independent analyses across 2 experienced statistical teams.

This study also has some limitations. Interpreting instrumental variable analysis is inherently challenging. The interpretation of instrumental variable effect estimates differs from that of conventional analyses. The undefined patient population in instrumental variable effect estimation makes it difficult to ascertain a mean treatment effect for an individual patient.

Potential residual confounding is another limitation, attributable to local practice variations associated with surgical thresholds, notwithstanding statistical adjustments, study method (instrumental variable analysis with a priori hypothesized neurosurgeon preferences), and robust effect estimates. Previous cluster analyses found no correlation between acute and critical care treatment options, including surgical treatment.^22^

TRACK-TBI patients were enrolled from US Level 1 trauma centers affiliated with academic institutions. Hence, our results may not be generalizable to centers with fewer institutional resources or different demographics.^49^

TRACK-TBI differed from CENTER-TBI in the following: no survey of predefined clinician preferences was completed, enrolled patients with TBI were less likely to have major extracranial injuries, and patients presenting with unsurvivable injuries as deemed by the TRACK-TBI site principal investigator were excluded from enrollment. In addition, patient-level effect estimates from regression and propensity score analyses differed from the center-level instrumental variable comparison in TRACK-TBI but not in CENTER-TBI, which may be attributable to the larger sample size of CENTER-TBI. The precise timing of clinical decision-making for medical and/or surgical management of TBI was not recorded in TRACK-TBI, constituting immortal time bias. However, we estimate this bias to be small because sensitivity analyses with different temporal definitions of acute surgery showed consistent ORs. Finally, estimating an overall effect of any intervention in TBI is amenable to a neutral result due to averaging heterogeneous effects.^33^

Future research should evaluate surgery in ASDH across age groups, the extent of neurologic compromise, and different medical comorbidity domains. We advocate for high-quality comparative studies specifically targeting patients likely to benefit from acute surgery in TBI with ASDH. Meta-analyses using patient-level data across similar large studies may possess the appropriate statistical strength to accommodate the required large samples.

## Conclusions

In this comparative effectiveness study, similar patients with traumatic ASDH received different acute surgical and conservative treatments across different centers due to center-specific factors. Outcomes were similar in centers preferring surgical evacuation and those preferring initial conservative treatment. For a patient with ASDH for whom a neurosurgeon finds clinical equipoise between acute surgery and initial conservative, nonsurgical management, initial conservative treatment may be considered.

## Notes

Supplement 1.
eMethods.

eResults.
**eFigure 1.** Directed Acyclic Graph of the Associations Motivating the Instrumental Variable Analysis **eTable 1.** Baseline Characteristics **eTable 2.** Admission and Treatment Characteristics **eTable 3.** Observed Treatment Proportions per Center **eTable 4.** Results of Sensitivity Analyses: Comparing Analytical Methods to Adjust for Confounding by Indication **eTable 5.** Hospital Course and Outcomes Across Centers with Different Preferences for Immediate Surgery of Acute Subdural Hematoma **eFigure 2.** Love Plot for the Effect of Weighing on Covariable Balance **eFigure 3.** Effects of Adjustment and Imputation on Between-Center Differences
eReferences.

Supplement 2. **Nonauthor Collaborators.** Transforming Research and Clinical Knowledge in Traumatic Brain Injury (TRACK-TBI) Study Investigators

Supplement 3.
Data Sharing Statement
