# Supplementary material for: Acute Surgery vs Conservative Treatment for Traumatic Acute Subdural Hematoma
Source: JAMA Netw Open. 2025 Oct 3;8(10):e2535200. doi: 10.1001/jamanetworkopen.2025.35200 (PMC12495496; doi:10.1001/jamanetworkopen.2025.35200)
Supplement: Supplement 1. — eMethods. eResults. eFigure 1. Directed Acyclic Graph of the Associations Motivating the Instrumental Variable Analysis eTable 1. Baseline Characteristics eTable 2. Admission and Treatment Characteristics eTable 3. Observed Treatment Proportions per Center eTable 4. Results of Sensitivity Analyses: Comparing Analytical Methods to Adjust for Confounding by Indication eTable 5. Hospital Course and Outcomes Across Centers with Different Preferences for Immediate Surgery of Acute Subdural Hematoma eFigure 2. Love Plot for the Effect of Weighing on Covariable Balance eFigure 3. Effects of Adjustment and Imputation on Between-Center Differences eReferences. [file jamanetwopen-e2535200-s001.pdf]

# Supplementary Online Content

Van Essen TA, Yue JK, Barber J, et al; Transforming Research and Clinical Knowledge in Traumatic Brain Injury (TRACK-TBI) Study Investigators. Acute surgery vs conservative treatment for traumatic acute subdural hematoma. *JAMA Netw Open.* 2025;8(10):e2535200. doi:10.1001/jamanetworkopen.2025.35200

**eMethods.**

**eResults.**

**eFigure 1.** Directed Acyclic Graph of the Associations Motivating the Instrumental Variable Analysis

**eTable 1.** Baseline Characteristics

**eTable 2.** Admission and Treatment Characteristics

**eTable 3.** Observed Treatment Proportions per Center

**eTable 4.** Results of Sensitivity Analyses: Comparing Analytical Methods to Adjust for Confounding by Indication

**eTable 5.** Hospital Course and Outcomes Across Centers with Different Preferences for Immediate Surgery of Acute Subdural Hematoma

**eFigure 2.** Love Plot for the Effect of Weighing on Covariable Balance

**eFigure 3.** Effects of Adjustment and Imputation on Between-Center Differences

**eReferences.**

This supplementary material has been provided by the authors to give readers additional information about their work.

© 2025 Van Essen TA et al. *JAMA Network Open.*

## eMethods.

The methodology and analysis strategy follow the CENTER-TBI study.[1,2] In summary, the outcomes were analyzed with respect to center treatment strategy (and not actual treatment) in instrumental variable (IV) analyses, a quasi-experimental approach. In this IV analysis, the IV center 'allocates' patients to be exposed to different likelihoods of receiving acute surgical evacuation. IV analysis is less biased by (unmeasured) confounding by indication and is the preferred analytical method in observational studies on acute neurosurgical decisions in traumatic brain injury.[3-5] The validity depends on 1) the extent to which the instrument is associated with the intervention under study, 2) the absence of independent association with the outcome under study, so that the IV is not associated with the measured or unmeasured patient health status, and 3) whether the instrument affects the outcome only through the treatment, not by any other pathways. We assessed the validity of these assumptions, although assumptions 2 and 3 are not testable formally.

To test assumption 1, the association of the instrument with acute surgery was modelled. We calculated adjusted probabilities of surgery with a fixed-effects logistic regression model with adjustment for confounders age, GCS, pupil reactivity, concomitant contusion and midline shift. This model was extended with random intercepts for center to estimate center effects on the probability of surgery. To quantify this regional surgical variation, the median odds ratio (MOR) was calculated which is a measure of treatment variation between centers that is not explained by other factors in the model or attributable to chance. The models with and without random-effects for center were compared with the likelihood ratio test to determine the significance of the between-center variation. Also, we included the partial F statistic as in our previous publication.[5,6]

Assumption 2 was evaluated by comparing the baseline prognosis, quantified by the CRASH-CT score, across the instrument. The CRASH-CT head injury model is a validated prognostic model based on the variables age, GCS, pupil reactivity to light, major extracranial injury, midline shift > 5mm, traumatic subarachnoid hemorrhage, and obliteration of the basal cisterns.[7] The IV was thereby checked according to recommendations.[4,5,6]

Assumption 3 (the exclusion criterion) has been addressed through various ways. First, the face validity of assumption 3 is reasonable. The concept is that patients are brought to hospitals to be treated by neurosurgeons whose treatment preference is unrelated to other local characteristics. Accidents occur independent of the surgical preference, as is the route an ambulance drive. We therefore included several center characteristics in Table 1 to facilitate assessment of baseline balance on instrument level, to appreciate whether center level confounding may exist. Second, while assumption 3 is indeed not testable, we did perform empirical justifications. We tested the assumption whether the effect of specific neurosurgical treatment choices/policies effects on outcome can be based on between-center variation without being substantially confounded by other treatments. We showed that it is was not possible to cluster hospitals with regard to specific treatment choices (intracranial pressure monitoring, coagulation and transfusion, neurosurgery, prophylactic antibiotics, and general ICU treatment policies).[8] Importantly, the absence of correlation between domains was most pronounced for neurosurgery. Thus, treatment preferences within a center are unrelated. This is in line with what would be expected, because treatment decisions are often made by different medical specialties. And third and last, the center coefficients from fixed-effects models, representing adjusted geographic treatment probabilities of acute surgery, were modelled in a random-effects ordinal regression with the aforementioned confounders as covariables. In this model, the random intercept represents the unexplained hospital effect (beyond all factors included in the model, including the instrument treatment preference) and should capture the measured and unmeasured hospital-level confounders, resulting in unbiased treatment effect estimates.

Initially in the primary analysis, only centers that enrolled at least 15 patients were included in the IV analyses, to minimize the influence of chance. That amounted to no change in the effect estimate (with 4 centers being discarded, and 17 patients). Therefore, we reported the analysis without exclusion of the small centers as our primary analysis.

*Sensitivity Analyses*

The primary analysis was replicated 1) without centers providing less than 15 patients (as mentioned), 2) without patients with unreactive pupils (poor prognosis) and patients with GCS 15 (excellent prognosis), and 3) with different definitions of acute surgery (A; all DHC or craniotomy < 24 hours as acute surgery (instead of 6 hours as cut-off), B; no time cut-off, all DHC or craniotomy for ASDH versus ASDH not surgically treated (ie, no DHC or craniotomy).

Additionally, we performed sensitivity analyses on patient-level. These additional analyses were performed with treatment defined at patient-level (exposed to intervention, yes/no), unadjusted, with multivariable regression and propensity score weighing (PSW). The propensity of being exposed to the intervention was computed using multivariable logistic regression with acute surgery as the dependent variable. PSW was used to balance the baseline prognosis of the surgical patients to the conservative patients. For the multivariable regression model, the aforementioned confounding variables of the primary analysis were considered independent variables. For the PSW we used a boosted regression algorithm for the weighing. We used random-effects models with center as the clustering variable for all patient-level analyses. These analyses were performed for the primary outcome on the complete cohort.

Secondary outcomes were analyzed with random-effects logistic and linear regression.

To assess the consistency of the estimate and the plausibility of proportionality of the OR, we present ORs for consecutive cut-offs on the GOSE.

© 2025 Van Essen TA et al. *JAMA Network Open.*

The main analyses were performed twice, by two independent statistical teams, following the proposed methodology from CENTER-TBI. The TRACK-TBI results are presented in the main manuscript. The CENTER-TBI team used R-software version 3.5.3 and RStudio version 1.1.463 with Multiple Imputation by Chained Equations (MICE) package (n=5) for missing data.

© 2025 Van Essen TA et al. *JAMA Network Open.*

## eResults.

*Quantification of practice variation and instrumental variable assumptions*

Repeating the MOR analysis with a random intercept showed an MOR of 2.74 (p=0.07), and with multiple imputation, an MOR of 2.22 (p=0.15) **(eFigure 1)**. MOR was 2.31 [p=0.16] for acute cranial surgery defined as surgical evacuation within 24 hours of first head CT (n=166). MOR was 2.00 [p=0.17] for cranial surgery defined as any surgical evacuation (n=191).

*Sensitivity analyses*

Repeating the main analysis for complete cases (patients whom have no missing values in baseline or outcome data) led to a similar effect estimate (OR=1.09 (95% CI: 0.87-1.38, n=417). In further sensitivity analyses, the association remained similar when excluding centers with less than 15 patients (OR=1.02 [0.84-1.24]). The results of other sensitivity analyses are described in the main manuscript and in **eTable 4**. The inverse probability weights did not need to be truncated/stabilized in the sensitivity center-level analysis. In the patient-level sensitivity analysis, we truncated the weighting at 9. Two subjects in the 'Acute' group had higher propensity weights than 9.

Both teams independently found similar results. The second analysis led to similar results as to the first. The first results, by the TRACK-TBI statistical team, are presented in the main manuscript.

The second analysis had similar findings: The instrument was strongly associated with the intervention under study with a MOR of 2.61 (p=0.002) and partial F statistic of 16 (assumption 1 of IV-analysis). Furthermore, the correlation between instrument and prognosis was small with Pearson's Rho correlation coefficient of 0.06 (assumption 2). Thus, the testable assumptions for IV analyses were met. The primary effect estimate (OR) of 1.09 [95% CI: 0.88-1.34] was in line with the other team's as well.

© 2025 Van Essen TA et al. *JAMA Network Open.*

**eFigure 1.** Directed Acyclic Graph of the Associations Motivating the Instrumental Variable Analysis

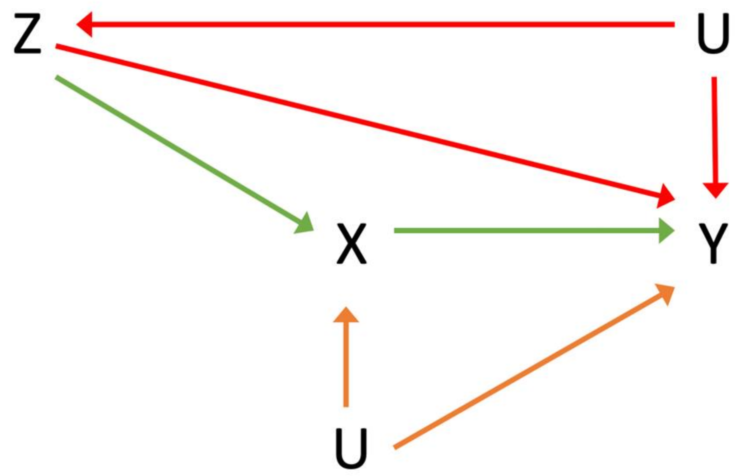


Z= instrument: center treatment preference
X= treatment: acute surgery
Y = outcome: 6-month Glasgow Outcome Scale Extended
U = confounder

Our conceptual instrument is a center's preference for acute surgery over (initial) conservative treatment for acute subdural hematoma. The assumed causal relations motivating the instrument are shown. The three assumptions of instrumental variable analysis: 1) Z has a causal effect on X; Neurosurgical centers vary in their preference for acute surgery and thus comparable patients are treated with acute surgery by some centers and with conservative treatment by other centers. 2) Z affects the outcome Y only through X; the direct arrow from preference to the outcome is explained by the arrows from Z through X to Y. The analogy is that centers predominantly influence functional outcome through the preferred type of immediate management. 3) No common causes for Z and Y (i.e. no confounding for effect Z on Y); no other pathways from Z to Y. This assumption will be satisfied if neurosurgical centers who favor acute surgery are not different than centers that favor conservative treatment. In summary, similar centers treat similar patient with different treatments (acute surgery or conservative management. The IV thereby 'allocates' patients to be exposed to different likelihoods of receiving acute surgical evacuation.

Of note, neurosurgical preference of a center is a variable that is not easily measured. We estimate a center's preference for acute surgery relative to conservative treatment by using the case-mix adjusted acute surgery proportion of that center.

© 2025 Van Essen TA et al. *JAMA Network Open.*

**eTable 1.** Baseline Characteristics

| | Total | ASDH treatment group | | | |
|---|---|---|---|---|---|
| | | **Acute** surgical evacuation within 6hr of CT | **Conservative** treatment | p [a] | p [b] |
| | | | | | |
| **Subjects** | **711** | **148** | **563** | | |
| | | | | | |
| **Age** | | | | | |
| Mean (SD) | 46.5 (19.4) | 43.2 (17.4) | 47.4 (19.8) | .024 | .006 |
| Median (IQR) | 46.5 (29.5 - 62.0) | 41 (28.5 - 55.8) | 47.5 (29.5 - 63.5) | | |
| Missing | 0 | 0 | 0 | | |
| | | | | | |
| **Sex** | | | | | |
| A) Male | 539 (76%) | 115 (78%) | 424 (75%) | .073 | .124 |
| B) Female | 172 (24%) | 33 (22%) | 139 (25%) | | |
| Missing | 0 | 0 | 0 | | |
| | | | | | |
| **Race** | | | | | |
| A) White | 562 (81%) | 117 (81%) | 445 (81%) | .009 | .014 |
| B) Black | 78 (11%) | 9 (6%) | 69 (13%) | | |
| C) Asian | 41 (6%) | 15 (10%) | 26 (5%) | | |
| D) Other | 12 (2%) | 4 (3%) | 8 (1%) | | |
| Missing | 18 (2%) | 3 (2%) | 15 (3%) | | |
| | | | | | |
| **Hispanic** | | | | | |
| No | 564 (81%) | 118 (81%) | 446 (81%) | .905 | .241 |
| Yes | 130 (19%) | 28 (19%) | 102 (19%) | | |
| Missing | 17 (2%) | 2 (2%) | 15 (3%) | | |
| | | | | | |
| **Education (Years)** | | | | | |
| Mean (SD) | 13.1 (3.3) | 12.5 (3.2) | 13.2 (3.3) | .135 | .002 |
| Median (IQR) | 12 (12-16) | 12 (12-14.3) | 12 (12-16) | | |
| <16 | 454 (73%) | 93 (78%) | 361 (72%) | .301 | .105 |
| ≥16 | 164 (27%) | 27 (23%) | 137 (28%) | | |
| Missing | 93 (13%) | 28 (19%) | 65 (12%) | | |
| | | | | | |
| **Married / Domestic Partnership** | | | | | |
| No | 347 (55%) | 76 (59%) | 271 (53%) | .235 | .839 |
| Yes | 288 (45%) | 52 (41%) | 236 (47%) | | |
| Missing | 76 (11%) | 20 (14%) | 56 (10%) | | |
| | | | | | |
| **Employed** | | | | | |
| No | 226 (36%) | 46 (37%) | 180 (36%) | .836 | .337 |
| Yes | 407 (64%) | 80 (63%) | 327 (64%) | | |
| Missing | 78 (11%) | 22 (15%) | 56 (10%) | | |
| | | | | | |
| **History of Cardiovascular Disorder** | | | | | |
| No | 523 (74%) | 118 (80%) | 405 (72%) | .060 | .008 |

© 2025 Van Essen TA et al. *JAMA Network Open.*

| | Total | ASDH treatment group | | | |
|---|---|---|---|---|---|
| | | Acute surgical evacuation within 6hr of CT | Conservative treatment | p [a] | p [b] |
| Yes | 188 (26%) | 30 (20%) | 158 (28%) | | |
| Missing | 0 | 0 | 0 | | |
| | | | | | |
| **History of Neurological Disorder** | | | | | |
| No | 628 (88%) | 128 (86%) | 500 (89%) | .472 | .561 |
| Yes | 83 (12%) | 20 (14%) | 63 (11%) | | |
| Missing | 0 | 0 | 0 | | |
| | | | | | |
| **History of Oncological Disorder** | | | | | |
| No | 682 (96%) | 144 (97%) | 538 (96%) | .484 | .629 |
| Yes | 29 (4%) | 4 (3%) | 25 (4%) | | |
| Missing | 0 | 0 | 0 | | |
| | | | | | |
| **History of Endocrine Disorder** | | | | | |
| No | 564 (79%) | 122 (82%) | 442 (79%) | .361 | .095 |
| Yes | 147 (21%) | 26 (18%) | 121 (21%) | | |
| Missing | 0 | 0 | 0 | | |
| | | | | | |
| **Population Type** | | | | | |
| 1 - Rural | 17 (3%) | 6 (5%) | 11 (2%) | .204 | .488 |
| 2 - Micropolitan | 22 (3%) | 5 (4%) | 17 (3%) | | |
| 3 - Metropolitan | 595 (94%) | 113 (91%) | 482 (95%) | | |
| Missing | 77 (11%) | 24 (4%) | 53 (9%) | | |
| | | | | | |
| **Cause of Injury** | | | | | |
| Road traffic incident | 327 (47%) | 74 (50%) | 253 (46%) | .414 | .228 |
| Incidental fall | 256 (36%) | 48 (33%) | 208 (37%) | | |
| Other non-intentional injury | 36 (5%) | 9 (6%) | 27 (5%) | | |
| Violence/assault | 55 (8%) | 8 (5%) | 47 (8%) | | |
| Suicide attempt | 0 (0%) | 0 (0%) | 0 (0%) | | |
| Other | 29 (4%) | 8 (5%) | 21 (4%) | | |
| Missing | 8 (1%) | 1 (1%) | 7 (1%) | | |
| | | | | | |
| **Injured by Acceleration/Deceleration** | | | | | |
| No | 447 (63%) | 86 (58%) | 361 (64%) | .182 | .022 |
| Yes | 264 (37%) | 62 (42%) | 202 (36%) | | |
| Missing | 0 | 0 | 0 | | |
| | | | | | |
| **Injured by Blow to Head** | | | | | |
| No | 522 (73%) | 107 (72%) | 415 (74%) | .754 | .679 |
| Yes | 189 (27%) | 41 (28%) | 148 (26%) | | |
| Missing | 0 | 0 | 0 | | |
| | | | | | |
| **Injured by Head to Object** | | | | | |
| No | 283 (40%) | 54 (36%) | 229 (41%) | .396 | .708 |
| Yes | 428 (60%) | 94 (64%) | 334 (59%) | | |
| Missing | 0 | 0 | 0 | | |
| | | | | | |

© 2025 Van Essen TA et al. *JAMA Network Open.*

| | Total | ASDH treatment group | | | |
|---|---|---|---|---|---|
| | | **Acute** surgical evacuation within 6hr of CT | **Conservative** treatment | p [a] | p [b] |
| **Injured by Ground-Level Fall** | | | | | |
| No | 547 (77%) | 116 (78%) | 431 (77%) | .742 | .217 |
| Yes | 164 (23%) | 32 (22%) | 132 (23%) | | |
| Missing | 0 | 0 | 0 | | |
| | | | | | |
| **Injured by Fall from Height** | | | | | |
| No | 514 (72%) | 103 (70%) | 411 (73%) | .411 | .183 |
| Yes | 197 (28%) | 45 (30%) | 152 (27%) | | |
| Missing | 0 | 0 | 0 | | |
| | | | | | |
| **Transferred from Other Hospital** | | | | | |
| No | 533 (75%) | 119 (80%) | 414 (74%) | .089 | .206 |
| Yes | 178 (25%) | 29 (20%) | 149 (26%) | | |
| Missing | 0 | 0 | 0 | | |
| | | | | | |
| **Alcohol Use** | | | | | |
| ≤2 drinks per day | 376 (63%) | 67 (60%) | 309 (64%) | .586 | .062 |
| >2 drinks per day | 221 (37%) | 44 (40%) | 177 (36%) | | |
| Missing | 114 (16%) | 37 (25%) | 77 (14%) | | |
| | | | | | |
| **Hypoxia** At presentation or in ED | | | | | |
| No | 647 (91%) | 122 (84%) | 525 (93%) | <.001 | .060 |
| Yes | 61 (9%) | 24 (16%) | 37 (7%) | | |
| Missing | 3 (0%) | 2 (1%) | 1 (0%) | | |
| | | | | | |
| **Hypotension** At presentation or in ED | | | | | |
| No | 641 (91%) | 125 (86%) | 516 (92%) | .027 | .055 |
| Yes | 67 (9%) | 21 (14%) | 46 (8%) | | |
| Missing | 3 (0%) | 2 (1%) | 1 (0%) | | |
| | | | | | |
| **Major Extracranial Injury** Any non-head/neck AIS ≥ 3 | | | | | |
| No | 550 (80%) | 101 (73%) | 449 (82%) | .017 | .008 |
| Yes | 136 (20%) | 38 (27%) | 98 (18%) | | |
| Missing | 25 (4%) | 9 (6%) | 16 (3%) | | |
| | | | | | |
| **Face AIS ≥ 3** | | | | | |
| No | 648 (98%) | 135 (97%) | 513 (98%) | .507 | <.001 |
| Yes | 14 (2%) | 4 (3%) | 10 (2%) | | |
| Missing | 49 (7%) | 9 (6%) | 40 (7%) | | |
| | | | | | |
| **Thorax/Chest AIS ≥ 3** | | | | | |
| No | 553 (84%) | 107 (77%) | 446 (85%) | .028 | .250 |
| Yes | 109 (16%) | 32 (23%) | 77 (15%) | | |
| Missing | 49 (7%) | 9 (6%) | 40 (7%) | | |
| | | | | | |
| **Abdomen/Pelvic AIS ≥ 3** Abdomen/Pelvic contents or pelvic girdle | | | | | |

© 2025 Van Essen TA et al. *JAMA Network Open.*

| | Total | ASDH treatment group | | | |
|---|---|---|---|---|---|
| | | **Acute** surgical evacuation within 6hr of CT | **Conservative** treatment | p [a] | p [b] |
| No | 648 (98%) | 136 (98%) | 512 (98%) | 1.000 | .741 |
| Yes | 14 (2%) | 3 (2%) | 11 (2%) | | |
| Missing | 49 (7%) | 9 (6%) | 40 (7%) | | |
| | | | | | |
| **Spine AIS ≥ 3**<br>Thoracic or Lumbar spine | | | | | |
| No | 657 (99%) | 138 (99%) | 519 (99%) | 1.000 | 1.000 |
| Yes | 5 (1%) | 1 (1%) | 4 (1%) | | |
| Missing | 49 (7%) | 9 (6%) | 40 (7%) | | |
| | | | | | |
| **Extremities AIS ≥ 3**<br>Upper or Lower | | | | | |
| No | 630 (95%) | 130 (94%) | 500 (96%) | .371 | .004 |
| Yes | 32 (5%) | 9 (6%) | 23 (4%) | | |
| Missing | 49 (7%) | 9 (6%) | 40 (7%) | | |
| | | | | | |
| **External AIS ≥ 3** | | | | | |
| No | 659 (100%) | 139 (100%) | 520 (99%) | 1.000 | .585 |
| Yes | 3 (0%) | 0 (0%) | 3 (1%) | | |
| Missing | 49 (7%) | 9 (6%) | 40 (7%) | | |
| | | | | | |
| **ED GCS Score** | | | | | |
| Mean (SD) | 10.5 (4.9) | 6.8 (4.4) | 11.4 (4.6) | <.001 | <.001 |
| Median (IQR) | 13 (6-15) | 5.5 (3-10) | 14 (8-15) | | |
| Mild (13-15) | 361 (54%) | 27 (20%) | 334 (62%) | <.001 | <.001 |
| Moderate (9-12) | 78 (12%) | 17 (12%) | 61 (11%) | | |
| Severe (3-8) | 234 (35%) | 94 (68%) | 140 (26%) | | |
| Missing | 38 (5%) | 10 (7%) | 28 (5%) | | |
| | | | | | |
| **ED GCS Motor Score** | | | | | |
| Mean (SD) | 4.4 (2.1) | 3.0 (2.1) | 4.8 (1.9) | <.001 | <.001 |
| Median (IQR) | 6 (2-6) | 2 (1-5) | 6 (4-6) | | |
| Missing | 43 (6%) | 13 (9%) | 30 (5%) | | |
| | | | | | |
| **Pupil Reactivity at ED Arrival**<br>Requires known values for both pupils | | | | | |
| Both reacting | 494 (81%) | 79 (59%) | 415 (87%) | <.001 | <.001 |
| One unreacting | 32 (5%) | 11 (8%) | 21 (4%) | | |
| Both unreacting | 84 (14%) | 43 (32%) | 41 (9%) | | |
| Missing/Incomplete | 101 (14%) | 15 (10%) | 86 (15%) | | |
| | | | | | |
| **Pre-Injury Medications** | | | | | |
| A - Neither | 533 (83%) | 109 (84%) | 424 (83%) | .078 | .124 |
| B - Anticoagulants only | 8 (1%) | 2 (2%) | 6 (1%) | | |
| C - Platelet inhibitors only | 92 (14%) | 15 (12%) | 77 (15%) | | |
| D - Both | 7 (1%) | 4 (3%) | 3 (1%) | | |
| Missing | 71 (10%) | 18 (12%) | 53 (9%) | | |
| | | | | | |
| **Time from Injury to Initial CT** | | | | | |
| Median (IQR) | 1.5 (1.0-2.7) | 1.3 (0.9-2.5) | 1.6 (1.0-2.7) | .112 | .263 |

© 2025 Van Essen TA et al. *JAMA Network Open.*

| | | ASDH treatment group | | | |
|---|---|---|---|---|---|
| | **Total** | **Acute** surgical evacuation within 6hr of CT | **Conservative** treatment | **p [a]** | **p [b]** |
| Missing | 0 | 0 | 0 | | |
| | | | | | |
| **Midline shift** | | | | | |
| Mean (SD) | 2.4 (4.5) | 8.0 (5.9) | 1.1 (2.9) | <.001 | <.001 |
| ≤5mm | 576 (83%) | 49 (37%) | 527 (94%) | <.001 | <.001 |
| >5mm | 120 (17%) | 84 (63%) | 36 (6%) | | |
| Missing | 15 (2%) | 15 (10%) | 0 | | |
| | | | | | |
| **CT Contusions** | | | | | |
| No | 307 (44%) | 41 (31%) | 266 (47%) | .001 | .026 |
| Yes | 389 (56%) | 92 (69%) | 297 (53%) | | |
| Missing | 15 (2%) | 15 (10%) | 0 | | |
| | | | | | |
| **CT SAH** | | | | | |
| No | 159 (23%) | 20 (15%) | 139 (25%) | .016 | .575 |
| Yes | 537 (77%) | 113 (85%) | 424 (75%) | | |
| Missing | 15 (2%) | 15 (10%) | 0 | | |
| | | | | | |
| **CT Cisterns** | | | | | |
| No effacement | 498 (72%) | 34 (26%) | 464 (82%) | <.001 | <.001 |
| Partial effacement | 127 (18%) | 52 (39%) | 75 (13%) | | |
| Complete effacement | 71 (10%) | 47 (35%) | 24 (4%) | | |
| Missing | 15 (2%) | 15 (10%) | 0 | | |
| | | | | | |
| **CT Marshall Score** | | | | | |
| 1 - DI I | 1 (0%) | 1 (1%) | 0 (0%) | <.001 | <.001 |
| 2 - DI II | 423 (61%) | 2 (2%) | 421 (75%) | | |
| 3 - DI III | 32 (5%) | 0 (0%) | 32 (6%) | | |
| 4 - DI IV | 22 (3%) | 0 (0%) | 22 (4%) | | |
| 5 - Evacuated mass lesion | 195 (28%) | 129 (98%) | 66 (12%) | | |
| 6 - Non-evacuated mass lesion | 17 (2%) | 0 (0%) | 17 (3%) | | |
| Missing | 21 (3%) | 16 (11%) | 5 (1%) | | |
| | | | | | |
| **CT Rotterdam Score** | | | | | |
| Mean (SD) | 3.2 (1.2) | 4.4 (1.4) | 2.9 (0.9) | <.001 | <.001 |
| Median (IQR) | 3 (2-4) | 5 (4-6) | 3 (2-3) | | |
| Missing | 15 (2%) | 15 (10%) | 0 | | |

Abbreviations: AIS, Abbreviated Injury Scale; ASDH, acute subdural hematoma; CT, computed tomography; DI, diffuse injury; ED, emergency department; GCS, Glasgow Coma Scale; IQR, interquartile range; SAH, subarachnoid hemorrhage; SD, standard deviation.

[a] Statistical significance by Mann-Whitney and Fisher's exact tests

[b] Statistical significance after reweighting the sample based on the group-allocation propensity score

© 2025 Van Essen TA et al. *JAMA Network Open.*

**eTable 2.** Admission and Treatment Characteristics

| | Total | ASDH treatment group | | p |
|---|---|---|---|---|
| | | **Acute** surgical evacuation within 6hr of CT (or prior) | **Conservative** treatment with surgery > 6hrs since CT (or never) | |
| | | | | |
| **Subjects** | **711** | **148** | **563** | |
| | | | | |
| **Surgery type** | | | | |
| Decompressive hemicraniectomy | 167 (75%) | 129 (88%) | - | -- |
| Craniotomy | 42 (19%) | 17 (12%) | - | |
| Missing/NA | | 2 | | |
| | | | | |
| **Secondary or delayed surgery** | | | | |
| Decompressive hemicraniectomy | 51 (7%) | 9 (6%) | 42 (7%) | |
| Craniotomy | 25 (4%) | 0 (0%) | 25 (4%) | |
| Acute subdural hematoma | 2 (0%) | 1 (1%) | 1 (0%) | |
| Epidural hematoma | 4 (1%) | 4 (3%) | 0 (0%) | |
| Intracerebral hematoma | 3 (0%) | 3 (2%) | 0 (0%) | |
| | | | | |
| **Time from CT to Surgery**<br>Among subjects who had surgery | | | | |
| Median (IQR) | 1.7 (0.7-5.1) | 1.1 (0.6-2.4) | 35 (13-101) | |
| | | | | |
| **Other surgery types** | | | | |
| Skull base repair | 5 (2%) | 0 (0%) | 5 (7%) | |
| Skull fracture repair | 3 (1%) | 0 (0%) | 3 (4%) | |
| CSF shunt | 1 (0%) | 1 (1%) | 0 (0%) | |
| Infection | 2 (0%) | 0 (0%) | 2 (0%) | |
| Ventriculostomy for CSF drainage | 1 (0%) | 0 (0%) | 1 (0%) | |
| Debridement – minimal | 1 (0%) | 0 (0%) | 1 (0%) | |
| Bone flap replacement | 2 (0%) | 2 (1%) | 0 (0%) | |
| Cranioplasty | 18 (3%) | 11 (7%) | 7 (1%) | |
| Other | 8 (1%) | 3 (2%) | 5 (1%) | |
| Missing/NA | 659 | 114 | 545 | |
| | | | | |
| **ICP monitor type** | | | | |
| None | 464 (65%) | 43 (29%) | 421 (75%) | <.001 |
| Ventriculostomy | 134 (19%) | 62 (42%) | 72 (13%) | |
| Intraparenchymal | 79 (11%) | 35 (24%) | 44 (8%) | |
| Both | 31 (4%) | 7 (5%) | 24 (4%) | |
| Other | 3 (0%) | 1 (1%) | 2 (0%) | |
| | | | | |
| **Average ICP**<br>Over entire hospital record | | | | |
| Mean (SD) | 14.0 (10.1) | 13.8 (10.5) | 14.2 (9.8) | .202 |
| Missing/NA | 466 | 45 | 421 | |
| | | | | |
| **Average CPP**<br>Over entire hospital record | | | | |
| Mean (SD) | 75.8 (13.8) | 76.2 (13.9) | 75.5 (13.8) | .276 |
| Missing/NA | 478 | 54 | 424 | |

© 2025 Van Essen TA et al. *JAMA Network Open.*

| | | | | |
|---|---|---|---|---|
| **Average TIL**<br>Over entire hospital record | | | | |
| Mean (SD) | 10.8 (4.4) | 12.2 (4.1) | 9.7 (4.3) | <.001 |
| Missing/NA | 500 | 55 | 445 | |
| | | | | |
| **Decision to withdraw active treatment** | | | | |
| No | 646 (91%) | 118 (80%) | 528 (94%) | |
| Yes | 65 (9%) | 30 (20%) | 35 (6%) | |
| | | | | |
| **Discharge location** | | | | |
| Other Hospital | 24 (4%) | 9 (6%) | 15 (3%) | <.001 |
| Rehabilitation unit | 157 (23%) | 43 (29%) | 114 (21%) | |
| Nursing home | 6 (1%) | 2 (1%) | 4 (1%) | |
| Home | 339 (50%) | 27 (18%) | 312 (59%) | |
| Other | 73 (11%) | 29 (20%) | 44 (8%) | |
| Died in hospital | 79 (12%) | 36 (25%) | 43 (8%) | |
| Missing | 33 | 2 | 31 | |
| | | | | |

Abbreviations: ASDH, acute subdural hematoma; CPP, cerebral perfusion pressure; CSF, cerebrospinal fluid; CT, computed tomography; DI, diffuse injury; ED, emergency department; GCS, Glasgow Coma Scale; ICP, intracranial pressure; IQR, interquartile range; SD, standard deviation; TIL, therapy intensity level.

© 2025 Van Essen TA et al. *JAMA Network Open.*

**eTable 3.** Observed Treatment Proportions per Center

| Subjects | Total | SDH Treatment Group | | |
|---|---|---|---|---|
| | | **Acute** surgical evacuation within 6hr of CT (or prior) | **Conservative** treatment with optional surgery > 6hrs since CT (or never) | **Acute Odds** |
| | | | | |
| **Site** | | | | |
| 1 | 138 | 22/138 (16%) | 116/138 (84%) | 0.19 |
| 2 | 44 | 7/44 (16%) | 37/44 (84%) | 0.19 |
| 3 | 125 | 39/125 (31%) | 86/125 (69%) | 0.45 |
| 4 | 43 | 12/43 (28%) | 31/43 (72%) | 0.39 |
| 5 | 15 | 0/15 (0%) | 15/15 (100%) | 0.00 |
| 6 | 15 | 0/15 (0%) | 15/15 (100%) | 0.00 |
| 7 | 133 | 31/133 (23%) | 102/133 (77%) | 0.30 |
| 8 | 20 | 2/20 (10%) | 18/20 (90%) | 0.11 |
| 9 | 28 | 1/28 (4%) | 27/28 (96%) | 0.04 |
| 10 | 42 | 5/42 (12%) | 37/42 (88%) | 0.14 |
| 11 | 28 | 5/28 (18%) | 23/28 (82%) | 0.22 |
| 12 | 45 | 8/45 (18%) | 37/45 (82%) | 0.22 |
| 13 | 1 | 0/1 (0%) | 1/1 (100%) | 0.00 |
| 14 | 4 | 1/4 (25%) | 3/4 (75%) | 0.33 |
| 15 | 3 | 1/3 (33%) | 2/3 (67%) | 0.50 |
| 16 | 2 | 0/2 (0%) | 2/2 (100%) | 0.00 |
| 17 | 18 | 8/18 (44%) | 10/18 (56%) | 0.80 |
| 18 | 7 | 6/7 (86%) | 1/7 (14%) | 6.00 |
| | | | | |
| **Total** | 711 | 148 (median 17%, IQR 5-27%) | 563 | |

© 2025 Van Essen TA et al. *JAMA Network Open.*

**eTable 4.** Results of Sensitivity Analyses: Comparing Analytical Methods to Adjust for Confounding by Indication

| Approach | Acute surgery (OR 95 % CI) | Number of patients (n) |
|---|---|---|
| **Unadjusted model** | 0.21 (0.14-0.30) | 517 |
| **Covariable adjustment in multivariable regression** [a] | 0.58 (0.33 - 1.01) | 517 |
| **Propensity score weighing** [b] | 0.49 (0.32 - 0.76) | 517 |
| | | |
| **Instrumental variable** [c] | | |
| Complete cases | 1.09 (CI 95% 0.87 - 1.38) | 417 |
| Multiple imputation | 1.06 (CI 95% 0.90 - 1.25) | 517 |
| Patients with both reactive pupils and GCS < 15 [d] | 0.99 (0.81 - 1.22) | 210 |
| With the cohort with centers < 15 patients excluded | 1.06 (0.88 - 1.29) | 506 |
| Adjusting with propensity score weighing (instead of regression adjustment) | 1.00 (0.79 - 1.27) | 517 |
| Additionally adjusted for cardiovascular preinjury comorbidity’ | 1.05 (0.88 - 1.26) | 517 |
| Additionally adjusted for anticoagulant use | 1.08 (0.94 - 1.24) | 517 |
| Additionally adjusted for timing injury to CT | 1.05 (0.88 - 1.26) | 517 |
| Additionally adjusted for transfer status | 1.06 (0.91 - 1.25) | 517 |
| Additionally adjusted for race | 1.04 (0.88 - 1.24) | 517 |
| Acute surgery defined as surgical evacuation within 24 hours of CT | 1.08 (0.82 - 1.43) | 517 |
| Acute surgery defined as surgical evacuation ever | 1.06 (0.80 - 1.39) | 517 |

Abbreviations: BTF, Brain Trauma Foundation; CI, confidence interval; GCS, Glasgow Coma Scale; OR, odds ratio.

All ORs represent the effect on the Glasgow Outcome Scale Extended estimated with proportional odds regression models with multiple imputation for missing covariates and inverse probability weighing for handling missing outcome data.

[a] Model was adjusted for the following confounders: age, GCS, pupillary reactivity, midline shift, hematoma size.

[b] For the propensity score weighing we used a boosted regression algorithm for the weighing.

[c] In these instrumental variable analyses, the adjusted common OR indicates the odds of a more favorable outcome for an increase from the 25th percentile to the 75th percentile of the range in exposure to the center’s preferences for acute surgery, adjusted for age, GCS, pupillary reactivity, midline shift, concomitant contusion and random hospital effect.

[d] This analysis is performed without patients with one or both unreactive pupil(s) (poor prognosis) and patients with GCS 15 (excellent prognosis).

© 2025 Van Essen TA et al. *JAMA Network Open.*

**eTable 5.** Hospital Course and Outcomes Across Centers With Different Preferences for Immediate Surgery of Acute Subdural Hematoma

| | **Acute surgery preference** (case-mix adjusted random-effects coefficient from mixed model) [a] | | | | | | |
|---|---|---|---|---|---|---|---|
| | **Quartile 1** (< -0.50) | **Quartile 2** (-0.50 to -0.25) | **Quartile 3** (-0.25 to +0.25) | **Quartile 4** (> +0.25) | **p** [b] | **r** [b] | **p** [c] |
| | | | | | | | |
| **Subjects** | **103** | **199** | **140** | **269** | | | |
| | | | | | | | |
| **Time from CT to (Acute) Surgery** | | | | | | | |
| Median (IQR) | 1.3 (0.4-4.7) | 1.3 (0.7-19.4) | 1.7 (1.1-3.8) | 1.7 (0.7-3.8) | <.01 | -0.16 | <.01 |
| | | | | | | | |
| **ICP Monitor Type** | | | | | | | |
| None | 76 (74%) | 122 (61%) | 112 (80%) | 154 (57%) | | | <.001 |
| Ventriculostomy | 18 (17%) | 61 (31%) | 6 (4%) | 49 (18%) | | | |
| Intraparenchymal | 7 (7%) | 12 (6%) | 16 (11%) | 44 (16%) | | | |
| Both | 2 (2%) | 3 (2%) | 5 (4%) | 21 (8%) | | | |
| Other | 0 (0%) | 1 (1%) | 1 (1%) | 1 (0%) | | | |
| | | | | | | | |
| **Average ICP** Over entire hospital record | | | | | | | |
| Mean (SD) | 13.5 (9.5) | 14.0 (6.3) | 15.3 (10.4) | 13.8 (12.1) | .42 | -0.05 | .02 |
| Missing/NA | 79 | 122 | 112 | 153 | | | |
| | | | | | | | |
| **Average CPP** Over entire hospital record | | | | | | | |
| Mean (SD) | 76.0 (14.2) | 73.8 (6.6) | 75.2 (12.1) | 77.4 (17.3) | .006 | 0.18 | <.001 |
| Missing/NA | 79 | 123 | 114 | 162 | | | |
| | | | | | | | |
| **Average TIL** Over entire hospital record | | | | | | | |
| Mean (SD) | 9.4 (5.3) | 10.9 (3.8) | 11.2 (4.8) | 10.7 (4.5) | .95 | 0.00 | .89 |
| Missing/NA | 92 | 135 | 116 | 157 | | | |
| | | | | | | | |
| **Average $PbrO_2$** Over entire hospital record | | | | | | | |
| Mean (SD) | 24.2 (8.8) | 31.4 (14.9) | 27.4 (16.7) | 25.5 (12.0) | .18 | -0.11 | .08 |
| Missing/NA | 98 | 151 | 124 | 178 | | | |
| | | | | | | | |
| **Hospital length of stay** | | | | | | | |
| Mean (SD) | 15.7 (19.5) | 13.6 (15.1) | 10.9 (13.4) | 13.9 (18.3) | .36 | -0.04 | .82 |
| Died / Missing | 26 | 23 | 21 | 42 | | | |
| | | | | | | | |
| **Died In-Hospital** | | | | | | | |
| No | 77 (79%) | 176 (93%) | 119 (90%) | 229 (87%) | | | .008 |
| Yes | 20 (21%) | 13 (7%) | 13 (10%) | 33 (13%) | | | |
| Missing | 6 | 10 | 8 | 7 | | | |
| | | | | | | | |
| **Decision to withdraw active treatment** | | | | | | | |
| Yes | 12 (12%) | 7 (4%) | 10 (7%) | 36 (13%) | | | .001 |
| | | | | | | | |
| **Discharge Destination** | | | | | | | |

© 2025 Van Essen TA et al. *JAMA Network Open.*

| | Acute surgery preference (case-mix adjusted random-effects coefficient from mixed model) [a] | | | | | | |
|---|---|---|---|---|---|---|---|
| | **Quartile 1** (< -0.50) | **Quartile 2** (-0.50 to -0.25) | **Quartile 3** (-0.25 to +0.25) | **Quartile 4** (> +0.25) | **p** [b] | **r** [b] | **p** [c] |
| A - Other Hospital | 1 (1%) | 3 (2%) | 0 (0%) | 20 (8%) | | | <.001 |
| B - Rehab Unit | 11 (11%) | 42 (22%) | 30 (23%) | 74 (28%) | | | |
| C - Nursing Home | 0 (0%) | 3 (2%) | 0 (0%) | 3 (1%) | | | |
| D - Home | 55 (57%) | 111 (59%) | 68 (52%) | 105 (40%) | | | |
| E - Other | 10 (10%) | 17 (9%) | 20 (15%) | 26 (10%) | | | |
| F - Died in Hospital | 20 (21%) | 13 (7%) | 13 (10%) | 33 (13%) | | | |
| Missing | 6 | 10 | 9 | 8 | | | |
| | | | | | | | |
| **6-Month GOSE** | | | | | | | |
| Mean (SD) | 4.3 (2.8) | 5.1 (2.3) | 5.5 (2.4) | 5.0 (2.5) | | | .07 |
| 1 | 21 (37%) | 18 (15%) | 15 (14%) | 43 (19%) | | | |
| 2 | 0 (0%) | 0 (0%) | 1 (1%) | 4 (2%) | | | |
| 3 | 4 (7%) | 23 (19%) | 10 (9%) | 30 (13%) | | | |
| 4 | 0 (0%) | 1 (1%) | 4 (4%) | 7 (3%) | | | |
| 5 | 6 (11%) | 12 (10%) | 12 (11%) | 28 (12%) | | | |
| 6 | 7 (12%) | 25 (20%) | 18 (17%) | 24 (10%) | | | |
| 7 | 12 (21%) | 27 (22%) | 21 (20%) | 47 (20%) | | | |
| 8 | 7 (12%) | 17 (14%) | 26 (24%) | 47 (20%) | | | |
| Missing | 46 | 76 | 33 | 39 | | | |

Abbreviations: ASDH, acute subdural hematoma; CPP, cerebral perfusion pressure; CSF, cerebrospinal fluid; CT, computed tomography; ED, emergency department; GCS, Glasgow Coma Scale; GOSE, Glasgow Outcome Scale Extended; ICP, intracranial pressure; IQR, interquartile range; SD, standard deviation; TIL, therapy intensity level.

[a] Treatment preference as defined by the case-mix adjusted probability of undergoing acute surgery (as opposed to initial conservative treatment) based on the observed acute surgery rates per center. The first category is less aggressive than the second and the second is less aggressive than the third and so forth. Importantly, the IV analysis used the acute surgery rates as continuous preference, the quartiles are presented for purposes of interpretability of baseline comparability. Based on random-effect coefficient from mixed model.

[b] Statistical significance and standardized effect-size by Spearman correlation based on the actual site-coefficient value (monotonicity assumption)

[c] Statistical significance by Kruskal-Wallis and Fisher's exact test (no monotonicity assumption)

© 2025 Van Essen TA et al. *JAMA Network Open.*

**eFigure 2.** Love Plot for the Effect of Weighing on Covariable Balance

Absolute unweighted and weighted standardized mean differences (SMDs) comparing the comparison groups of the primary analysis on the listed variables.

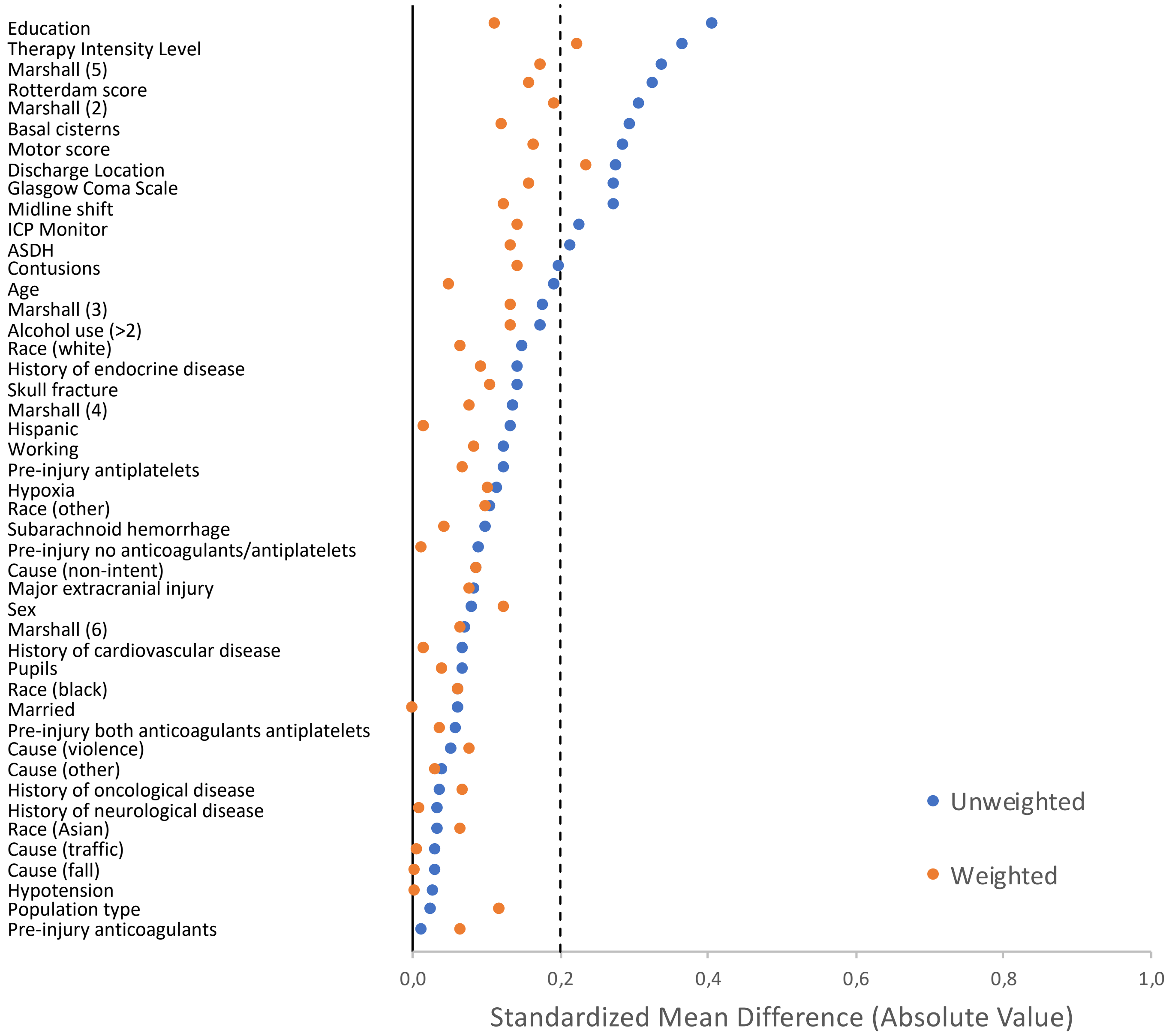


© 2025 Van Essen TA et al. *JAMA Network Open.*

**eFigure 3.** Effects of Adjustment and Imputation on Between-Center Differences

**Panel A.** The x-axis presents the log odds of the adjusted acute surgery rates per center. A logistic random-effects model was used to estimate acute surgery preference per center with corresponding 95% CIs. **Panel B.** Similar to A (a logistic random-effects model), except additionally adjusted for the predefined confounders age, GCS, pupil reactivity, concomitant contusion and midline shift, with multiple imputation for the missing variables (see **eMethods**).

**A**

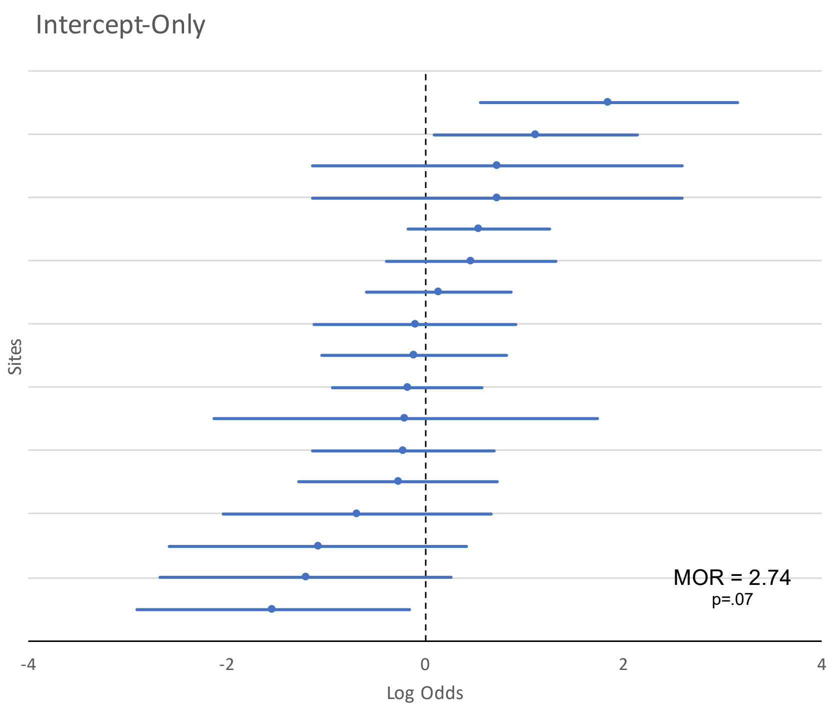


**B**

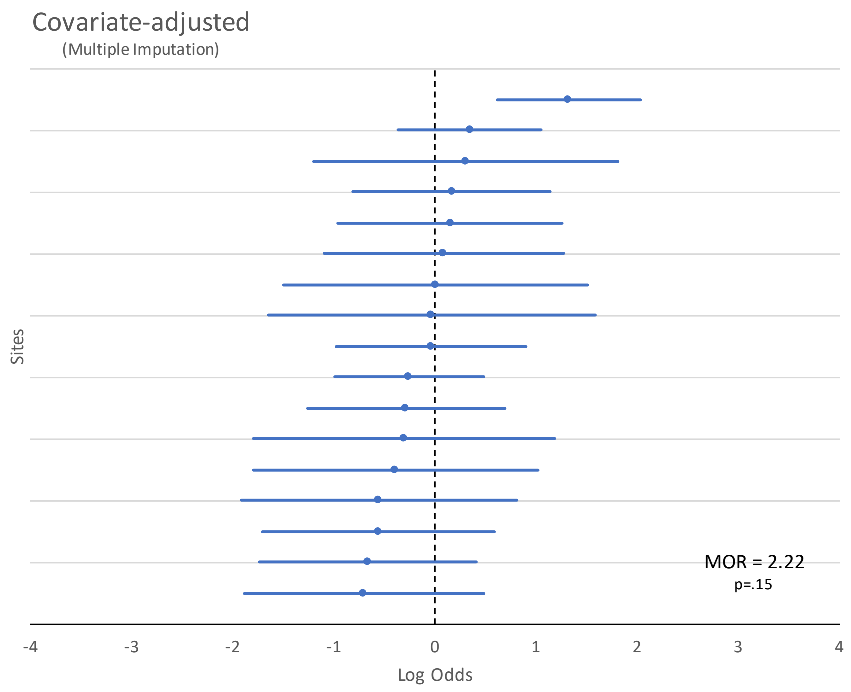


© 2025 Van Essen TA et al. *JAMA Network Open.*

## eReferences.

1 Maas AIR, Menon DK, Steyerberg EW, *et al.* Collaborative European NeuroTrauma Effectiveness Research in Traumatic Brain Injury (CENTER-TBI): a prospective longitudinal observational study. *Neurosurgery* 2015; **76**: 67–80.
2 van Essen TA, Volovici V, Cnossen MC, *et al.* Comparative effectiveness of surgery in traumatic acute subdural and intracerebral haematoma: study protocol for a prospective observational study within CENTER-TBI and Net-QuRe. *BMJ Open* 2019; **9**: e033513–8.
3 Stukel TA, Fisher ES, Wennberg DE, Alter DA, Gottlieb DJ, Vermeulen MJ. Analysis of observational studies in the presence of treatment selection bias: effects of invasive cardiac management on AMI survival using propensity score and instrumental variable methods. *JAMA* 2007; **297**: 278–85.
4 Brookhart MA, Rassen JA, Schneeweiss S. Instrumental variable methods in comparative safety and effectiveness research. *Pharmacoepidem Drug Safe* 2010; **19**: 537–54.
5 Cnossen MC, van Essen TA, Ceyisakar IE, *et al.* Adjusting for confounding by indication in observational studies: a case study in traumatic brain injury. *Clinical Epidemiology* 2018; **10**: 841–52.
6 Davies NM, Smith GD, Windmeijer F, Martin RM. Issues in the reporting and conduct of instrumental variable studies: a systematic review. *Epidemiology* 2013; **24**: 363–9.
7 MRC CRASH Trial Collaborators. Predicting outcome after traumatic brain injury: practical prognostic models based on large cohort of international patients. *BMJ* 2008; **336**: 425–9.
8 Ceyisakar IE, Huijben JA, Maas AIR, et al. Can We Cluster ICU Treatment Strategies for Traumatic Brain Injury by Hospital Treatment Preferences? *Neurocrit Care*. 2022 Jun; **36**(3): 846-856.

© 2025 Van Essen TA et al. *JAMA Network Open.*
