# Supplementary material for: Acute Surgery vs Conservative Treatment for Traumatic Acute Subdural Hematoma
Source: JAMA Netw Open. 2025 Oct 3;8(10):e2535200. doi: 10.1001/jamanetworkopen.2025.35200 (PMC12495496; doi:10.1001/jamanetworkopen.2025.35200)
Supplement: Supplement 2. — Nonauthor Collaborators. Transforming Research and Clinical Knowledge in Traumatic Brain Injury (TRACK-TBI) Study Investigators [file jamanetwopen-e2535200-s002.pdf]

Supplemental Online Content: Nonauthor Collaborators
*First name, last name, and suffix (if applicable) are required and will appear in PubMed.

***Group Name(s): TRACK-TBI Investigators**

***First Name and Middle Initial(s)**	***Last Name**	***Suffix (eg, Jr, III)**	Academic Degrees	Institution	Location (city, state/province, country)	Role or Contribution, eg, chair, principal investigator	Group (if more than 1 Group listed in the byline) and/or Subgroup (eg, Steering Committee)
Shawn R.	Eagly		PhD	Department of Neurological Surgery, University of Pittsburgh	Pittsburgh, Pennsylvania, USA	Collaborator	
Mahmoud M.	Elguindy		MD, PhD	Department of Neurological Surgery, University of California, San Francisco	San Francisco, California, USA	Collaborator	
Leila L.	Etemad		BA	Department of Neurological Surgery, University of California, San Francisco	San Francisco, California, USA	Collaborator	
Brian	Fabian		MPA	Department of Neurological Surgery, University of California, San Francisco	San Francisco, California, USA	Project manager	
Christine J.	Gotthardt		MS	Department of Neurological Surgery, University of California, San Francisco	San Francisco, California, USA	Collaborator	
Sonia	Jain		PhD	Biostatistics Research Center, Herbert Wertheim School of Public Health and Longevity Science, University of California San Diego	San Diego, California, USA	Collaborator	
Frederick K.	Korley		MD, PhD	Department of Emergency Medicine, University of Michigan	, Ann Arbor, Michigan, USA	Collaborator	
Vijay	Krishnamoorthy		MD	Department of Anesthesiology, Duke University	Raleigh, North Carolina, USA	Collaborator	
Christine	Mac Donald		PhD	Department of Neurological Surgery, University Washington	Seattle, Washington, USA	Collaborator	
Ramesh	Grandhi		MD	Department of Neurological Surgery, University of Utah Medical Center	Salt Lake City, Utah, USA	Collaborator	

Supplemental Online Content: Nonauthor Collaborators
*First name, last name, and suffix (if applicable) are required and will appear in PubMed.

***First Name and Middle Initial(s)**	***Last Name**	***Suffix (eg, Jr, III)**	Academic Degrees	Institution	Location (city, state/province, country)	Role or Contribution, eg, chair, principal investigator	Group (if more than 1 Group listed in the byline) and/or Subgroup (eg, Steering Committee)
Randall	Merchant		PhD	Department of Anatomy, Virginia Commonwealth University	Richmond, Virginia, USA	Collaborator	
Pratik	Mukherjee		MD, PhD	Department of Radiology and Biomedical Imaging, University of California, San Francisco	San Francisco, California, USA	Collaborator	
Laura B.	Ngwenya		MD, PhD	Department of Neurological Surgery, University of Cincinnati	Cincinnati, Ohio, USA	Collaborator	
Ava M.	Puccio		PhD	Department of Neurological Surgery, University of Pittsburgh	Pittsburgh, Pennsylvania, USA	Collaborator	
Gabriela G.	Satris		MSN, MSc	Department of Neurological Surgery, University of California, San Francisco	San Francisco, California, USA	Collaborator	
David M.	Schnyer		PhD	Department of Psychology, University of Texas at Austin	Austin, Texas, USA	Collaborator	
Xiaoying	Sun		MS	Biostatistics Research Center, Herbert Wertheim School of Public Health and Longevity Science	San Diego, California, USA	Collaborator	
Sabrina R.	Taylor		PhD	Department of Neurological Surgery, University of California, San Francisco	San Francisco, California, USA	Collaborator	
Mary J.	Vassar		MS	Department of Neurological Surgery, University of California, San Francisco	San Francisco, California, USA	Collaborator	
Ross D.	Zafonte		DO	Department of Rehabilitation Medicine	Harvard Medical School, Boston, Massachusetts, USA	Collaborator	
