# Supplementary material for: Acute Surgery vs Conservative Treatment for Traumatic Acute Subdural Hematoma
Source: JAMA Netw Open. 2025 Oct 3;8(10):e2535200. doi: 10.1001/jamanetworkopen.2025.35200 (PMC12495496; doi:10.1001/jamanetworkopen.2025.35200)
Supplement: Supplement 3. — Data Sharing Statement [file jamanetwopen-e2535200-s003.pdf]

Data Sharing Statement

Van Essen. Acute Surgery vs Conservative Treatment for Traumatic Acute Subdural Hematoma. *JAMA Netw Open*. Published October 03, 2025. doi:10.1001/jamanetworkopen.2025.35200

Data
Data available: Yes
Data types: Deidentified participant data, Data dictionary
How to access data: Listed items available through barber@uw.edu after approval by data protection officer team.
When available: With publication

Supporting Documents
Document types: Statistical/analytic code, Informed consent form
How to access documents: Upon reasonable request through essen@lumc.nl
When available: With publication

Additional Information
Who can access the data: Approved researchers
Types of analyses: For a prespecified purpose
Mechanisms of data availability: After approval of a proposal
